# Endoscopically assessed mucus parameters in equine asthma: Relationship to clinical history and cytological findings data

**DOI:** 10.1111/evj.70002

**Published:** 2025-07-24

**Authors:** Julia Drespling, Leonie Berwanger, Heike Kühn, Bianca Schwarz, Marcus Doherr, Lars Mundhenk

**Affiliations:** ^1^ Institute of Veterinary Pathology, Freie Universität Berlin Berlin Germany; ^2^ EquiZyt UG Laboratory Steinhöring Germany; ^3^ Institute of Veterinary Epidemiology and Biostatistics, Freie Universität Berlin Berlin Germany

**Keywords:** bronchoalveolar lavage fluid, horse, mucus quantity, mucus scoring, respiratory cytology, tracheobronchial secretions, schleim menge, schleimbeurteilung, bronchoalveoläre lavageflüssigkeit, tracheobronachiale sekrete, respiratorische zytologie, pferd

## Abstract

**Background:**

Mucus parameters are hallmark diagnostic features of equine asthma (EA).

**Objectives:**

To investigate the relationship between mucus quantity score and mucus viscosity score with signalment, history, clinical findings and cytological parameters.

**Study Design:**

Retrospective cross‐sectional study.

**Methods:**

Mucus quantity and viscosity scores, signalment, history and clinical findings recorded for diagnostic purposes from up to 1599 samples, and cytological values of the corresponding bronchoalveolar lavage fluid (BALF) and tracheobronchial secretions were analysed. The cut‐off value of a mucus quantity score >2 for the diagnosis of mild to moderate EA (MEA) was used to calculate the odds ratios for MEA and severe EA (SEA). Associations were tested via a multivariate Kruskal–Wallis test. The mucus scores were analysed pairwise with cytological characteristics, anamnestic data and clinical signs by testing for independence using the chi‐square test. Correlations were evaluated using the non‐parametric Spearman's rank correlation coefficient.

**Results:**

A mucus quantity score >2 was associated with increased odds of diagnosing SEA (odds ratio 3.6, 95% confidence interval 1.75, 7.17, *p* < 0.001), but not for MEA. A weak positive correlation was found between neutrophil granulocytes in BALF and mucus quantity score (rho: 0.353, *p* < 0.001) as well as mucus viscosity score (rho: 0.225, *p* < 0.001). The mucus scores for quantity and viscosity were significantly associated (*p* < 0.001) with age, respiratory rate and arterial oxygen partial pressure values, and significantly associated (*p* < 0.001) with season, type of nasal discharge and respiratory pattern. However, no associations were found with body condition score (BCS), sex and breed.

**Main Limitations:**

This was a convenience sample with a cytologically unremarkable control group, but no healthy control group.

**Conclusions:**

Mucus quantity score is a diagnostic parameter for the diagnosis of SEA. Associations between mucus scores and neutrophilic influx and with some clinical parameters were identified, while there was no confirmed relationship to BCS.

## Zusammenfassung

**Hintergrund:**

Schleimparameter sind charakteristische Merkmale und indikative diagnostische Komponenten von Equinem Asthma (EA).

**Ziel der Studie:**

Querschnittsstudie zur Untersuchung der Verhähltnisse zwischen dem Mucus Quantity Score (m.q.) und dem Mucus Viscosity Score (m.v.) und Signalement, Anamnese, klinischen Befunden und zytologischen Parametern.

**Studiendesign:**

Retrospektive Querschnittsstudie.

**Methodik:**

Analysiert wurden die Schleimmenge und Viskositätswerte, das Signalement, die Anamnese und klinischen Befunde, die zu Diagnosezwecken von bis zu 1599 Proben erfasst wurden, sowie die zytologischen Werte der entsprechenden bronchoalveolären Lavagflüssigkeit (BALF) und tracheobronchialen Sekrete (TBS). Der Cut‐off‐Wert eines Mucus Quantity Score >2 für die Diagnose eines leichten bis mittelschweren EA (MEA) wurde für die Berechnung der Odds Ratios für MEA und schweres EA (SEA) verwendet.

Assoziationen wurden mittels eines multivariaten Kruskall‐Wallis‐Tests geprüft. Die Mucus‐Scores wurden paarweise mit zytologischen Merkmalen, anamnestischen Daten und klinischen Symptomen analysiert, indem die Unabhängigkeit mit dem Chi‐Square‐Test geprüft wurde. Korrelationen wurden mit dem nichtparametrischen Spearman‐Rangkorrelationskoeffizienten bewertet.

**Ergebnisse:**

Ein Mucus Quantity Score > 2 war mit einer 3,6‐fachen [OR 3,6, 95% 1,75, 7,17, *p* < 0,001] statistisch signifikant erhöhten Wahrscheinlichkeit verbunden, ein SEA zu diagnostizieren, aber nicht für ein MEA. Eine schwache positive Korrelation wurde zwischen neutrophilen Granulozyten in der BALF und des Mucus Quantity Score (rho: 0,353; *p* < 0,001) sowie dem Mucus Viscosity Score (rho: 0,225; *p* < 0,001) festgestellt. Darüber hinaus waren die Schleimwerte Mucus Quantity Score und Mucus Viscosity Score signifikant (*p* < 0,001) mit Alter, Atemfrequenz und arteriellen Sauerstoffpartialdruckwerten sowie signifikant (*p* < 0,001) mit Jahreszeit, Art des Nasenausflusses und Atmungsmuster verbunden. Es wurden jedoch keine Zusammenhänge mit dem Body Condition Score (BCS), dem Geschlecht und der Rasse festgestellt.

**Haupteinschränkungen:**

Es handelte sich um eine Zufallsstichprobe mit einer “zytologisch unauffälligen” Kontrollgruppe, aber ohne eine gesunke Kontrollgruppe.

**Schlussfolgerung:**

Der Mucus Quantity Score ist ein diagnosticher Parameter für die Diagnose von SEA. Aossiziationen zwischen Mucus‐Scores und neutrophilem Einstrom sowie manchen klinischen Parametern wurden identifiziert, während eine Beziehung zum BCS nicht bestätigt werden konnte.

## 1 INTRODUCTION

Excessive mucus quantity is a hallmark of equine asthma (EA) which includes the mild to moderate EA (MEA) (also known as inflammatory airway disease or IAD) and severe equine asthma (SEA) (formerly known as recurrent airway obstruction or RAO).^1^ Mucus is an important factor in protecting the airways in terms of humidifying and warming the inhaled air, the function of the cilia and the mechanical defence of the host.^2^, ^3^ However, excessive mucus or its stasis can have adverse effects and contribute to the pathogenesis of various airway diseases in mammals, such as mucoviscidosis, alias cystic fibrosis, asthma and chronic obstructive pulmonary disease in humans^3^, ^4^ and EA in horses.^5^, ^6^, ^7^ The mechanism and the factors that affect mucus accumulation are poorly understood.^8^

Endoscopic scoring of mucus quantity in the region of the tracheobronchial tree, in addition to history, clinical presentation and airway cytology, is indispensable in the diagnosis of EA.^1^, ^9^, ^10^ Several studies have shown that mucus quantity is positively correlated to varying degrees with the proportion of neutrophils in bronchoalveolar lavage fluid (BALF).^8^, ^11^, ^12^, ^13^ In other studies, the amount of tracheal mucus was not correlated to the BALF neutrophil percentage, in particular in young horses with mild clinical signs.^14^, ^15^, ^16^, ^17^ Mucus quantity was negatively correlated to the mast cell and macrophage percentages in the BALF.^12^ The quantity of tracheal mucus has been weakly positively correlated with age.^12^ Inconsistent results have been found regarding the link to the thickness of the tracheal septum: Koch et al.^18^ found no correlation to mucus quantity score, while Koblinger et al.^12^ demonstrated at least a weak positive correlation between the two parameters. Cough is one of the main symptoms of EA and has been associated with the detection of tracheal mucus in Thoroughbred racehorses.^19^, ^20^ Nasal discharge is considered an indicator of increased mucus quantity.^20^ However, data concerning the relationship between mucus quantity score in horses and parameters such as sex, breed and body condition score (BCS) are lacking. In humans, BCS and sex are known to be associated with diseases with mucus accumulation. Increased BCS was found to be associated with an increased risk of EA.^21^ The prevalence of asthma in adult women is significantly higher than in men.^22^, ^23^ The influence of the menstrual cycle, pregnancy and sex steroid hormones in general can increase the occurrence and severity of asthma exacerbations.^24^ In particular, oestrogen dehydrates the surface of the airways, which changes the composition of mucus and promotes airway plugging.^24^

The viscoelastic property of the mucus influences pathophysiology and is altered in disease. Viscoelasticity is determined by varying composition of water, mucin, glycoproteins, lipids, cells and their degradation products and serum proteins.^25^ In human asthma, for example, the filamentous actin released by neutrophils increased the viscoelasticity of the mucus.^26^ Dyscrinia with changes in the gel‐sole layer of the epithelial cells has also been described in SEA.^27^, ^28^ There are various grading systems for endoscopic assessment of apparent mucus viscosity.^11^, ^29^, ^30^ However, data on the relationship between mucus viscosity and cytology, medical history and clinical signs are lacking. Experimentally, rheological mucus properties did not correlate with BALF cytology; however, viscoelasticity increased during environmental challenge in SEA.^25^

In the current study, the relationship between mucus quantity score and mucus viscosity score in horses and cytological parameters, signalment, history and clinical findings was comprehensively analysed. We hypothesised that (1) the severity of EA would correlate with mucus quantity score and mucus viscosity score; (2) the cut off value of mucus quantity score >2 offers a higher chance of diagnosing MEA in sport and pleasure horses and that the chance is different from SEA; (3) different scores of mucus quantity score and mucus viscosity score are associated with a different cell profile in BALF cytology and tracheobronchial secretions (TBS); (4) the percentage of differentiated cells correlates with mucus quantity score and mucus viscosity score; (5) the detection of foreign particles (pollen, spores, hyphae) is associated with mucus quantity score and mucus viscosity score; (6) there are associations between age, sex, breed, BCS and the season of sampling with mucus quantity score and mucus viscosity score; (7) nasal discharge and respiratory pattern are associated with mucus quantity score and mucus viscosity score and (8) coughing, respiratory rate, swelling of tracheal septum and arterial partial pressure of oxygen correlate with mucus quantity score and mucus viscosity score.

## 2 MATERIALS AND METHODS

### 2.1 Study design

Between January 2022 and February 2023, 2039 BALF and/or TBS samples were sent from various equine practices and clinics to EquiZyt UG laboratory in Steinhöring, Germany, for diagnostic purposes. In advance, the veterinarians received a standardised clinical history and examination questionnaire (Survey S1). The BALF were collected with two boluses of 250 mL of sterile sodium chloride solution in all cases. The collected fluid was centrifuged at 300–500*g*, a smear was prepared and air dried. The TBS samples were taken by direct aspiration of tracheal mucus where possible, smeared on slides and air dried. The smears and, if possible, remaining BALF were sent to the laboratory within 2–3 days. To preserve good cell quality, the BALFs were cooled during transport and partially treated with stabiliser (isopropyl alcohol, ethyl alcohol, EDTA).

The questionnaire was divided into an owner and a veterinary component. Age, breed, sex and coughing (none, mild, moderate, severe) and history of fever were requested from the owner. On the day of sampling, BCS from 1 to 9,^31^ the presence and quality of nasal discharge (none, serous, seromucous, mucous, mucopurulent, purulent, bloody), the respiratory pattern (normal/abnormal), the respiratory rate and, if available, the partial pressure of oxygen (pO_2_ value) of the arterial blood gas analysis should be answered by the veterinarians. The mucus quantity and mucus viscosity scores and presence of swelling at the tracheal septum (also known as the tracheal bifurcation or carina) were noted. Veterinarians were instructed in the endoscopy scoring system by means of a detailed information sheet (Data S1). Mucus quantity score was recorded according to Gerber et al.^11^ with values from 0 to 5 (Table S1), mucus viscosity score with values from 0 to 3 (Table S2) and the swelling of the tracheal septum with values from 0 to 3 (Table S3).^29^ The season was later defined according to the date of sampling as winter (December–February), spring (March–May), summer (June–August) and autumn (September–November). To the best of our knowledge, each horse was only included once in the study.

### 2.2 Cytology

For cytological evaluation, the air‐dried BALF and TBS smears were stained using the Diff‐Quick staining technique (RAL Diff‐QuikTM; CellaVision RAL Diagnostics) for cell counting and toluidine blue solution (Toluidine Blue1% aqueous; Morphisto) for mast cell counts.^32^ If the quality of the smears was moderate, new smears were prepared from BALF, compared with the smears sent in and counted from the slide with the best quality. If only fluid was sent in, the BALF was centrifuged at 300–500*g* for 10 min, the pellet subsequently smeared on a slide, dried and stained as described above. From each smear, 500 cells were counted and the percentages of neutrophil granulocytes, eosinophil granulocytes, alveolar macrophages, lymphocytes and mast cells were calculated. The percentage of mast cells was verified using toluidine staining. In addition, foreign particles such as pollen, fungal spores and conidia as well as fragments of hyphae were recorded as present or absent.

Horses were classified into four groups based on BALF cytology^1^: samples with >25% neutrophil granulocytes in the BALF were assigned to the SEA group and those with between 10% and 25% neutrophil granulocytes, >5% eosinophil granulocytes and/or >5% mast cells were assigned to the MEA group. Horses with neutrophils ≤5%, eosinophils ≤1% and mast cells ≤2% were classified as ‘cytologically unremarkable’. Samples with neutrophils >5% and ≤10%, mast cells >2% and ≤5% and eosinophils >1% and ≤5% were assigned to a ‘not defined’ category and excluded from the odds ratios analysis (Figure S1). In the analysis of the correlation between the severity of the diagnosis and mucus parameters, the group was included. Samples from 73 horses with fever and/or with cytologically detectable intracellular bacteria were excluded in the analysis (Figure S1).

### 2.3 Data analysis

Only horses in which data on mucus quantity and/or viscosity were analysed further (Figure S1). Data analyses were performed using the statistical software IBM SPSS Statistics version 29.0.0.0 (241). The association between a mucus quantity score 2 and the diagnosis of MEA and SEA^1^ was explored with a chi‐square test. In order to explore whether different mucus quantity scores and mucus viscosity scores were associated with different cell compositions, the multivariate Kruskal–Wallis test was performed with samples for which both BALF and TBS were available because the values were not normally distributed. The effect size for each cell type was reported using ETA Squared values derived from the corresponding univariate Kruskal–Wallis tests. The calculated effect sizes allow us to compare how strongly each of the different cell type counts are affected by changes in the mucus scores. As a degree of strength of impact, they are, however, to be viewed with caution, because the one‐on‐one univariate Kruskal–Wallis tests do not account for interactions between the cell types which are definitely present. In order to assess how mucus scores are related to other diagnostic parameters, secretion was analysed pairwise with cytological characteristics, clinical history data and clinical findings by testing for independence using the chi‐square test. If at least one of the expected cell frequencies was below 5, Fisher's exact test was used. The numerical linear correlations between the scores for mucus quantity and viscosity and cell counts, age, cough, respiratory rate, tracheal septum swelling and arterial pO_2_ value were evaluated using the non‐parametric Spearman's rank correlation coefficient (Spearman's rho). Using Cohen's classification, values between rho = 0.1 and rho = 0.3 are categorised as weak, between rho = 0.3 and rho = 0.5 as moderate and rho >0.5 as strong correlation.^33^ A varying number of valid entries in the data table accounted for differing sample sizes for each statistical test applied. The significance level for all tests was set at *p* < 0.05. We used the Bonferroni correction to correct for multiple testing. The significance level for the association analyses within the different groups was set to *p* < 0.003 for the cytology analyses, *p* < 0.01 for the anamnestic data and *p* < 0.008 for the clinical signs.

## 3 RESULTS

Of the 2039 samples, mucus quantity score was available in 1599 cases and mucus viscosity score was available in 1238 cases. Samples were derived from 84 different equine clinics and practices. The clinic with the largest number of samples submitted 120 samples. Diagnosis based on BALF analysis was made in 1234 cases with mucus quantity data and 949 cases with mucus viscosity data (Table 1).

**TABLE 1:** Number of data available for analysis of relationships between mucus quantity and mucus viscosity scores and clinical and cytological findings in equine asthma cases.

	No. of samples available for analysis of mucus quantity relationships	No. of samples available for analysis of mucus viscosity relationships	Type of relationship analysis
Cytological data			
TBS samples	1370	1061	Spearman rho
BAL samples	1234	948	Spearman rho
Of which BAL and TBS both available	1011	774	Multivariate Kruskal–Wallis test
Of which only BAL available	223	174	
Of which only TBS available	359	287	
Diagnosis	1234	948	Spearman rho
Clinical findings and signalment			
Age	1499	1166	Spearman rho
Sex	1564	1212	Chi‐square
Breed	1485	1157	Chi‐square
Body condition score	279	275	Spearman rho
Season of sampling	1557	1208	Chi‐square
Cough	1146	902	Spearman rho
Nasal discharge	1014	804	Chi‐square
Respiratory pattern	1504	1169	Chi‐square
Respiratory rate	1365	1082	Spearman rho
Tracheal septum swelling	1212	967	Spearman rho
Arterial pO_2_	422	364	Spearman rho

### 3.1 Relationship mucus parameters and diagnosis (Hypotheses 1 and 2)

**FIGURE 1:**
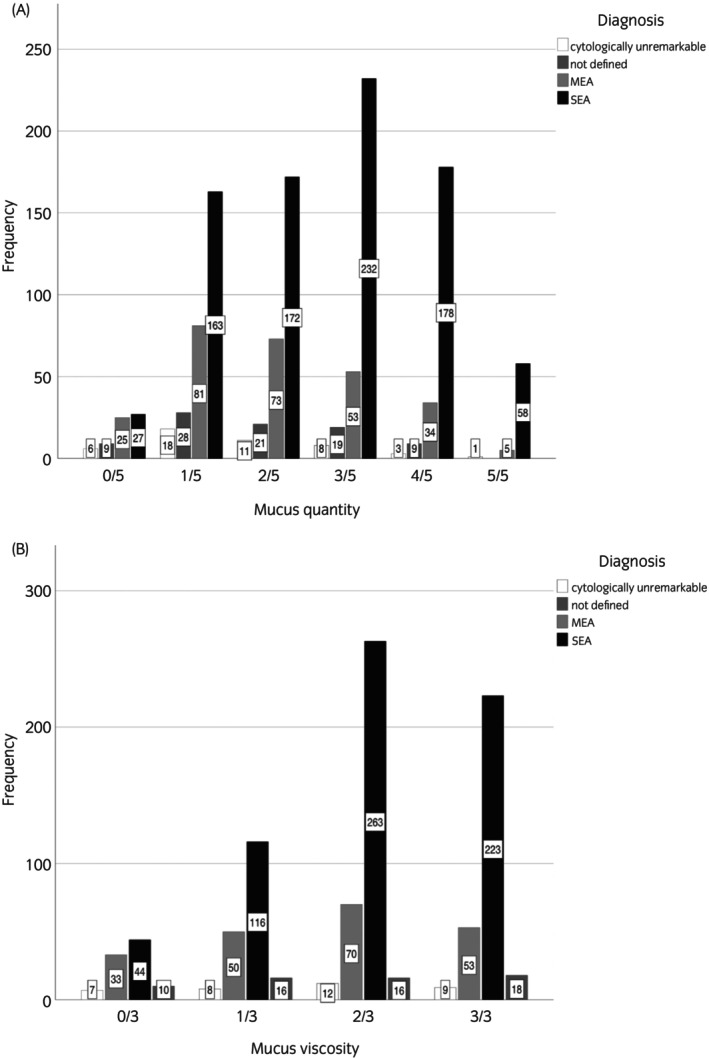
Distribution of diagnosis in relation to mucus quantity score (A) and mucus viscosity score (B).

The diagnoses of EA based on cytology cell counts showed a positive, weak correlation between the severity of disease and the mucus quantity score (*n* = 1234, rho = 0.251, *p* < 0.001) and mucus viscosity score (*n* = 948, rho = 0.151, *p* < 0.001). SEA was diagnosed approximately three times more often than MEA. SEA was diagnosed more frequently with a mucus quantity score ≥2, while MEA was identified more frequently with a mucus quantity score ≤2 (Figure 1).

Mucus quantity score >2 was associated with the diagnosis of SEA (odds ratio [OR] 3.6, 95% confidence interval [CI] 1.75, 7.17) but not MEA (OR 1.4, 95% CI 0.67, 2.92).

### 3.2 Relationship between mucus parameters and cytological components (Hypotheses 3 and 4)

There were significant differences in the composition of cells in cytology between different mucus quantity score (*W* ^2^ = 797.123, df = 60, *p* < 0.001) and mucus viscosity score (*W* ^2^ = 788.565, df = 36, *p* < 0.001; Hypothesis 3; Table 2). Post hoc analysis of the variance for the different cell types revealed the largest effect size for neutrophil granulocytes for mucus quantity score (BALF: 0.127 [*p* < 0.001]; TBS: 0.231 [*p* < 0.001]; Table 2) and mucus viscosity score (BALF: 0.063 [*p* < 0.001]; TBS: 0.080 [*p* < 0.001]; Table 2). BALF mast cells and eosinophil granulocytes had no significant association with mucus quantity score and mucus viscosity score in BALF (Table 2). In TBS, there was no significant relationship between mucus quantity and mucus viscosity scores and mast cells. A significant association between eosinophil granulocytes and mucus quantity score was found (effect size 0.011 [*p* = 0.01]; Table 2). There were greater effect sizes across all cell types in TBS than in BALF. The effect sizes were also higher for mucus quantity score compared to mucus viscosity score.

**TABLE 2:** Pairwise Kruskal–Wallis tests for mucus quantity score and mucus viscosity score and different cell populations in equine asthma.

Dependent variable	Test statistic *H*	Effect size	*p*‐value	Bonferroni‐corrected *p*‐value
Mucus quantity score				
Macrophages BALF	117.471	0.092	<0.001	<0.01
Lymphocytes BALF	85.070	0.065	<0.001	<0.01
Neutrophil granulocytes BALF	160.679	0.127	<0.001	<0.01
Mast cells BALF	12.798	—	0.03	0.3
Eosinophil granulocytes BALF	4.606	—	0.5	—
Macrophages TBS	301.364	0.217	<0.001	<0.01
Lymphocytes TBS	209.171	0.150	<0.001	<0.01
Neutrophil granulocytes TBS	319.529	0.231	<0.001	<0.01
Mast cells TBS	6.578	—	0.3	—
Eosinophil granulocytes TBS	19.761	0.011	0.001	0.01
Mucus viscosity score				
Macrophages BALF	51.010	0.051	<0.001	<0.01
Lymphocytes BALF	31.487	0.030	<0.001	<0.01
Neutrophil granulocytes BALF	62.030	0.063	<0.001	<0.01
Mast cells BALF	4.107	—	0.3	—
Eosinophil granulocytes BALF	5.258	—	0.2	—
Macrophages TBS	75.806	0.069	<0.001	<0.01
Lymphocytes TBS	68.987	0.062	<0.001	<0.01
Neutrophil granulocytes TBS	87.772	0.080	<0.001	<0.01
Mast cells TBS	1.372		0.7	—
Eosinophil granulocytes TBS	4.822		0.2	—

*Note*: *p*‐values were adjusted following the Bonferroni correction.

BALF macrophage and lymphocyte proportions had a weak negative correlation with mucus quantity score (macrophage: *n* = 1234, rho: −0.299, *p* < 0.001; lymphocyte: *n* = 1234, rho: −0.248, *p* < 0.001) and mucus viscosity score (macrophage: *n* = 948, rho: −0.219, *p* < 0.001; lymphocyte: *n* = 948, rho: −0.143, *p* < 0.001) (Figure S2). Neutrophil granulocytes were moderately positively correlated with mucus quantity score (*n* = 1234, rho: 0.353, *p* < 0.001) and weakly positively correlated with mucus viscosity score (*n* = 948, rho: 0.225, *p* < 0.001), respectively, in the BALF. The percentage of mast cells and eosinophil granulocytes in BALF did not correlate significantly with either of the mucus parameters.

These findings were largely confirmed in the TBS (Figure S3). The proportion of macrophages (*n* = 1370, rho: −0.463, *p* < 0.001) and lymphocytes (*n* = 1370, rho: −0.385, *p* < 0.001) in the TBS moderately correlated negatively with mucus quantity score, while the correlation with mucus viscosity score was weakly negative (macrophage: *n* = 1061, rho: −0.254, *p* < 0.001; lymphocyte: *n* = 1061, rho: −0.242, *p* < 0.001). The BALF neutrophil percentage correlated positively with mucus quantity score (*n* = 1370, rho: 0.477, *p* < 0.001) and mucus viscosity score (*n* = 1061, rho: 0.272, *p* < 0.001). In TBS, a weak negative correlation was shown between mucus quantity score and eosinophil granulocytes (*n* = 1370, rho: −0.107, *p* < 0.001); however, there was no significant correlation between eosinophil granulocytes and mucus viscosity score or between mast cells and both scores.

### 3.3 Relationship between mucus parameters and detection of foreign particles (Hypothesis 5)

**FIGURE 2:**
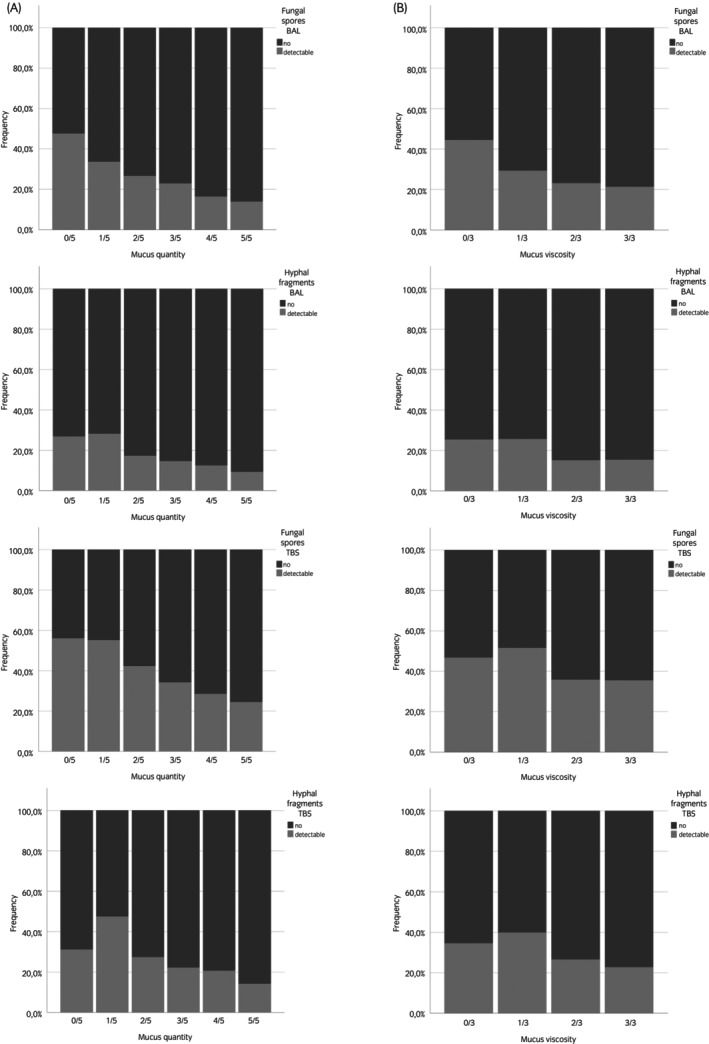
Association between foreign particles and mucus quantity (A) and viscosity (B) scores. Significant associations were detected between fungal spores in BALF and TBS and mucus quantity score (*p* < 0.001) and mucus viscosity score (*p* < 0.001). Increasing mucus quantity and viscosity scores were associated with a decrease in the detection of fungal spores in BALF and TBS. Detection of hyphal fragments in BAL and TBS was significantly associated with a decrease in mucus quantity score (*p* < 0.001) and mucus viscosity score (*p* < 0.001) of mucus. The graphs were scaled to 100%. BALF, bronchoalveolar lavage fluid; TBS, tracheobronchial secretions.

Significant associations were found between the detection of fungal spores and mucus quantity and mucus viscosity scores in BALF (mucus quantity score: *n* = 1234, *p* < 0.001; mucus viscosity score: *n* = 948, *p* < 0.001) as well as TBS (mucus quantity score: *n* = 1370, *p* < 0.001; mucus viscosity score: *n* = 1061, *p* < 0.001). Also, a significant association was found between hyphal fragments and mucus parameters in both BALF (mucus quantity score: *n* = 1234, *p* < 0.001; mucus viscosity score: *n* = 1234, *p* < 0.001) and TBS (mucus quantity score: *n* = 1370, *p* < 0.001; mucus viscosity score: *n* = 1061, *p* < 0.001). Lower values of both mucus parameters were associated with the detection of fungal spores and hyphal fragments in BALF and TBS (Figure 2). No significant relationship between mucus quantity and mucus viscosity scores and the detection of pollen was shown in either BALF (mucus quantity score: *n* = 1234, *p* = 0.4; mucus viscosity score: *n* = 948, *p* = 0.2) or TBS (mucus quantity score: *n* = 1370, *p* = 0.02; mucus viscosity score: *n* = 1061, *p* = 0.5).

### 3.4 Relationship between mucus parameters and signalment and clinical history (Hypothesis 6)

Increasing age was correlated with increased mucus quantity (*n* = 1499, rho: 0.190, *p* < 0.001; Figure S4) and mucus viscosity (*n* = 1166, rho: 0.097, *p* < 0.001) scores. However, no significant correlation was found between BCS and the mucus parameters (mucus quantity score: *n* = 279, *p* = 0.7; mucus viscosity score: *n* = 275, *p* = 0.9). Also, neither breed (mucus quantity score: *n* = 1485, *p* = 0.3; mucus viscosity score: *n* = 1157, *p* = 0.06) nor sex (mucus quantity score: *n* = 1564, *p* = 0.1; mucus viscosity score: *n* = 1212, *p* = 0.8) was associated with both mucus parameter scores.

**FIGURE 3:**
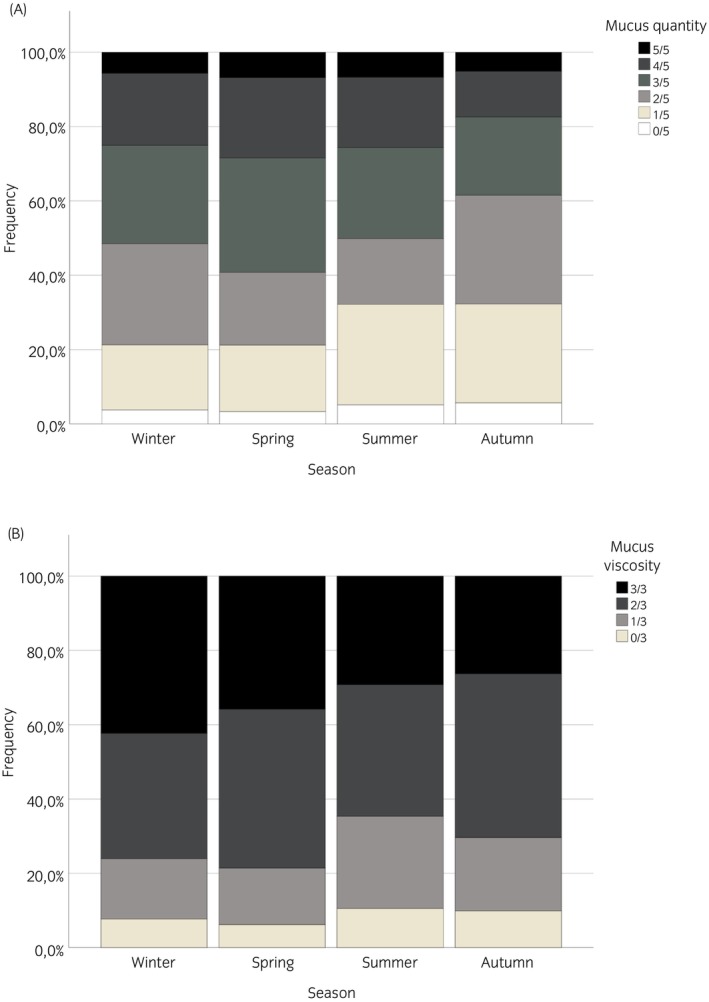
Associations between season and mucus quantity (A, *p* < 0.001) and mucus viscosity (B, *p* < 0.01). Mucus quantity score >2 was found in 60% of cases during spring, while during autumn mucus quantity score >2 was recorded in only 38% of horses. Mucus viscosity score (>1/3) were detected most frequently in winter and least frequently in summer. The graphs were scaled to 100%.

Season was associated with both mucus quantity score (*n* = 1557, *p* < 0.001) and mucus viscosity score (*n* = 1208, *p* < 0.001). Overall, the lowest number of submitted samples (*n* = 251) was found in autumn and the highest sample volume, with more than twice as many samples, was recorded in spring (*n* = 664). During spring, a mucus quantity score >2 was found in 60% of cases, while during autumn a mucus quantity score >2 was recorded in only 38% (Figure 3). In line with this, a significant association between season and diagnosis was shown. The proportion of SEA cases was detected highest in spring at 70%, while in autumn only 57% of samples were indicative of SEA. Viscous mucus with a mucus viscosity score of >1 was most frequently detected in winter (76% of horses) and least frequently detected in summer (65% of horses) (Figure 3).

### 3.5 Relationship between mucus parameters and clinical findings (Hypotheses 7 and 8)

**FIGURE 4:**
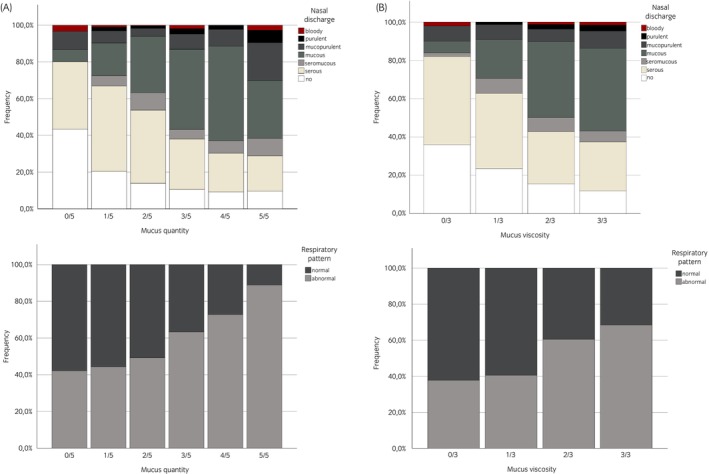
Association between mucus quantity (A) and viscosity (B) scores and nasal discharge and respiratory pattern. A significant association was detected between the type of nasal discharge and mucus quantity score (*p* < 0.001) and mucus viscosity score (*p* < 0.001). Increasing mucus quantity and viscosity scores was associated with an increase in the percentage of mucous and mucopurulent nasal discharge. Pathological respiratory pattern was significantly associated with an increase in mucus quantity score (*p* < 0.001) and mucus viscosity score (*p* < 0.001) of mucus. The graphs were scaled to 100%.

Both mucus parameters were significantly associated with type of nasal discharge (mucus quantity score: *n* = 1014, *p* < 0.001; mucus viscosity score: *n* = 804, *p* < 0.001). An increase in mucus quantity and mucus viscosity scores was associated with an increase in the percentage of mucus and mucopurulent nasal discharge (Figure 4). An abnormal respiratory pattern was associated with mucus parameters (mucus quantity score: *n* = 1504, *p* < 0.001; mucus viscosity score: *n* = 1169, *p* < 0.001). For severity of coughing, a significant, weak positive correlation was found with the mucus quantity score (*n* = 1146, rho: 0.259, *p* < 0.001; Figure S5); however, there was no significant correlation with mucus viscosity score (*n* = 902, *p* = 0.04). Consistently, respiratory rate was also weakly positively correlated with the mucus quantity score (*n* = 1365, rho: 0.196, *p* < 0.001) and the mucus viscosity score (*n* = 1082, rho: 0.138, *p* < 0.001). The swelling of the tracheal septum was also partially recorded as part of routine endoscopic examination. The swelling of the tracheal septum was moderately or weakly positively correlated with mucus quantity score (*n* = 1212, rho: 0.328, *p* < 0.001; Figure S5) or mucus viscosity score (*n* = 967, rho: 0.243, *p* < 0.001; Figure S5). The arterial partial pressure of oxygen showed a moderate negative correlation with mucus quantity score (*n* = 422, rho: −0.349, *p* < 0.001; Figure S5). The correlation with mucus viscosity score was less pronounced (*n* = 364, rho: −0.207, *p* < 0.001; Figure S5).

## 4 DISCUSSION

Increased mucus quantity and viscosity are characteristic features of EA that can be recorded in the course of clinical examination. A mucus quantity score ≥2 is a typical feature of MEA in sports and pleasure horses.^1^ Horses in this study with a mucus quantity score >2 were indeed more likely to be diagnosed with SEA. Our results were inconclusive with regard to MEA. Based on our data, the cut‐off value and its diagnostic interpretation should thus be critically scrutinised. In addition, other diseases such as bacterial, viral, fungal or parasitic pneumonia, which are also associated with mucus accumulation, should be ruled out when interpreting the mucus quantity score in relation to asthma. Nevertheless, mucus quantity score and mucus quantity score showed a positive correlation with the severity of the diagnosis (Hypothesis 1) in line with Meiseberg et al.,^34^ who also showed this relationship. However, mucus quantity score per se is not specific and should only be interpreted in combination with clinical history, clinical and cytological findings. Additionally, it should be noted that in this study, the categorisation of the diagnosis was based exclusively on cytology and the healthy control group only included cytologically unremarkable horses. This could have led to bias in the calculation of the odds as well as the correlation. Furthermore, it could not be ruled out that severe asthmatics in remission were among the sampled horses. Another limitation of our classification is the lack of inclusion of clinical signs such as increased respiratory effort in the distinction between MEA and SEA. In a previous study, respiratory distress was used as the sole distinguishing feature between IAD and RAO.^35^ The authors critically discussed that there is also a larger proportion of horses that did not show respiratory distress at rest and nevertheless corresponded to the cytological and endoscopic findings of a SEA horse.^35^ BALF cytology is considered the gold standard for the diagnosis of EA. In contrast to clinical findings, cytological differentiation between subtypes is based on a clear classification scheme.^36^

The relationship between both mucus scores and different cytological, clinical and anamnestic parameters was also analysed. Overall, the correlations shown between the respective mucus score and other parameters such as neutrophils were stronger for mucus quantity score than for mucus viscosity score (Hypothesis 4). Both scores are recorded visually, and it has been shown that the validated mucus quantity score, which is commonly used clinically and also in research, is the most reliable and meaningful, which is confirmed by our data.^11^ In contrast, it had been demonstrated that a visually assessed mucus viscosity score did not correlate with the measured physical property of viscoelasticity, and data obtained should be interpreted with caution.^11^ In the current study, a slightly modified viscosity score was used and almost all correlations and associations could be demonstrated with both mucus scores; however, with different effect sizes. Certain correlations were found only for mucus quantity score, but no relationship was found only for mucus viscosity score. Mucus viscosity, which impairs mucociliary clearance, can contribute to obstruction.^30^, ^37^

The strongest positive correlation was found between mucus quantity score and the number of neutrophilic granulocytes (Hypothesis 4). This had been demonstrated in several studies before, and the correlation coefficient calculated in this study is slightly below the values published by Gerber et al.^11^ and Koblinger et al.^12^ In contrast, other studies have found no correlation between mucus accumulation and the increase in neutrophils.^14^, ^15^, ^16^, ^17^ The driving forces of neutrophil influx into airways and mucus accumulation are still largely unknown in EA. However, neutrophils produce potent secretory substances such as elastase and oxidising radicals, which can lead to hypersecretion.^38^, ^39^ The positive correlation between mucus quantity score and neutrophils is in line with the suggestion that neutrophils play a role in airway mucus accumulation. However, it should also be considered that both events are independent consequences of lower airway inflammation.

The mucus scores did not correlate with mast cell percentage (Hypothesis 4), in line with a previous study.^16^ Only one previous study revealed a negative weak correlation between mast cell percentage and mucus accumulation.^12^ However, altogether the data argue against a role for this cell type in mucus quantity. The biological relevance of the weak, negative correlation between mucus quantity score and percentage of eosinophils in the TBS found in the current study should be interpreted with caution (Hypothesis 4) and it was not demonstrated in BALF. A possible explanation might be that the effect sizes were higher in TBS than in BALF for all cell types. Further studies on mucus scores and cytological parameters in horses with eosinophil‐ and mast cell‐rich phenotype are needed.

Fungal elements such as spores and hyphae are ubiquitous in the environment of horses, and these elements are suspected to trigger EA.^40^ Horses with fungal elements identified on tracheal wash (TW) cytology were twice as likely to be diagnosed with MEA.^41^ However, it was shown recently that the presence of fungal elements in the TW was not associated with EA.^42^ The verification of our Hypothesis 5 showed that increasing mucus quantity and mucus viscosity scores were significantly associated with a decrease in the detection of fungal elements in BALF and TBS. Since mucous nasal discharge was associated with increasing mucus scores (Hypothesis 7), it is plausible that inhaled particles such as fungal spores or hyphae are trapped in the upper respiratory tract before reaching the lower airways. The association between an increase in tracheal mucus scores and the detection of mucous nasal discharge also suggests that the nasal mucus might arise from the lower airways (Hypothesis 7). It is also possible that the increased mucus quantity leads to increased capacity for capture and improved removal of foreign particles and thus to less detection of spores and hyphae in TBS and BALF.

Further clinical findings such as coughing, abnormal respiratory pattern, respiratory rate and arterial pO_2_ values (Hypotheses 7 and 8) were also associated or correlated with both mucus scores and are in line with previous reports.^8^, ^20^, ^30^, ^43^, ^44^ In airway disease, excessive mucus or its impaired clearance can cause mucus plugging. This, in addition to bronchoconstriction, leads to these clinical signs and there is a reported association between increased mucus accumulation and poor racing performance.^45^ In particular, the association of mucus quantity score and mucus viscosity score with breathing pattern and the negative correlation with arterial pO_2_ values suggest an increased capacity for diffuse luminal obstruction of the lower airways.

Contradictory results had been reported for the correlation of the swelling of the tracheal septum and the mucus quantity score. One study showed no relationship,^18^ whereas another study could demonstrate such a relationship.^12^ Our results of the analysis of our hypothesis 8 support a correlation between mucus accumulation and tracheal septum swelling. However, this could also be independent consequences of inflammation of the lower airways.

We have shown a positive correlation with age (Hypothesis 6).^12^, ^43^ However, neither mucus quantity nor viscosity scores were associated or correlated with sex, breed or BCS (Hypothesis 6). A BCS ≥7 has been suspected as a risk factor for EA^21^ and obesity is a recognised risk factor for asthma in humans.^46^, ^47^, ^48^, ^49^ Obese human asthmatics have severe and more frequent exacerbations and a worse response to therapy.^49^ However, we did not demonstrate any relationship between BCS and mucus quantity or viscosity scores.

Our sample submission numbers were lowest in autumn and the frequency of high mucus quantity scores also decreased in autumn, while both were markedly higher in spring (Hypothesis 6). To date, only one other study has investigated the relationship between seasonality and mucus production in a population of healthy animals, with a significantly higher amount of tracheal mucus found in November than in May.^50^ These contrasting results may be explained by different study populations and by climatic differences between Denmark and Germany.

These data were not collected specifically for this study but were part of the routine clinical procedure of the examination. Consequently, in the absence of a clinically healthy control group, ‘cytologically unremarkable’ horses were used as a control group. This could have biased the analyses, as the horses were examined on the basis of indications of respiratory diseases. However, some were investigated for poor performance with a range of possible non‐respiratory diagnoses.^51^, ^52^

We used questionnaire‐based data. The clinical examination and sampling for diagnostic purposes were carried out by different veterinarians, which may lead to variation. However, all veterinarians were trained equine veterinarians and only data based on a standardised, comparable protocol were considered. In addition, it is known that owners can provide more reliable information about the severity/frequency of coughing in horses with MEA compared to veterinarians.^53^ Therefore, these parameters were collected from the owners. Nevertheless, incomplete questionnaires were another limitation; however, the group sizes were more than sufficient to analyse all questions. Medication could also have an effect on mucus quantity and viscosity, as well as on the cytological and clinical examination and was not taken into account in our study.

In summary, this study identified an association between the mucus quantity score and disease severity in a large cohort of horses, which supports the potential utility of the score. Important correlations between mucus scores and neutrophilic influx or clinical parameters were emphasised, and the hypothesised relationship with BCS could not be confirmed.

## FUNDING INFORMATION

This study was supported by the German Research Council (Deutsche Forschungsgemeinschaft MU 3015/3‐1).

## CONFLICT OF INTEREST STATEMENT

The authors declare no conflicts of interest.

## AUTHOR CONTRIBUTIONS

**Julia Drespling:** Conceptualization; investigation; writing – original draft; formal analysis; data curation; visualization. **Leonie Berwanger:** Writing – original draft; formal analysis; conceptualization. **Heike Kühn:** Project administration; resources; writing – review and editing. **Bianca Schwarz:** Project administration; resources; writing – review and editing. **Marcus Doherr:** Writing – review and editing; supervision. **Lars Mundhenk:** Writing – review and editing; supervision; funding acquisition; conceptualization.

## DATA INTEGRITY STATEMENT

Julia Drespling and Lars Mundhenk had full access to all the data in the study and take responsibility for the integrity of the data and the accuracy of the data analysis.

## ETHICAL ANIMAL RESEARCH

Research ethics committee oversight not currently required by this journal: procedures were performed as part of clinical investigations.

## INFORMED CONSENT

Explicit owner consent for inclusion of animals in this study was not obtained. Owners/trainers were made aware that case information may be used for research in general.

## Supporting information


**Data S1.** Information sheet.

**Figure S1.** Flow chart of the analyses and the samples used for them.

**Figure S2.** Correlation between BALF cell proportions and mucus quantity and viscosity.

**Figure S3.** Correlation between TBS cell proportions and mucus quantity and viscosity.

**Figure S4.** Correlation between mucus quantity/viscosity and age.

**Figure S5.** Correlation between mucus quantity/viscosity and cough, respiratory frequency, swelling of tracheal septum, and pO_2_ value of arterial blood gas analysis.

**Survey S1.** Examination questionnaire.

**Table S1.** Scoring of mucus quantity.

**Table S2.** Scoring of mucus viscosity.

**Table S3.** Scoring of swelling of tracheal septum.

## Data Availability

The data that support the findings of this study are available upon reasonable request from the corresponding author. Open data sharing exemption granted by the editor.
