# Supplementary material for: Endoscopically assessed mucus parameters in equine asthma: Relationship to clinical history and cytological findings data
Source: Equine Vet J. 2025 Jul 24;58(3):767–78. doi: 10.1111/evj.70002 (PMC13041601; doi:10.1111/evj.70002)
Supplement: Supplementary file 1 — Data S1. Information sheet. [file EVJ-58-767-s007.pdf]

Data S1: Information sheet.

* **Body-Condition-Score**

1 Poor	Animal extremely emaciated. Spinous processes, ribs, tailhead, tuber coxae and ischii projecting prominently. Bone structure of withers, shoulders and neck easily noticeable. No fatty tissue can be felt
2 Very thin	Animal emaciated. Slight fat covering over base of spinous processes, transverse processes of lumbar vertebrae feel rounded. Spinous processes, ribs, tailhead, tuber coxae and ischii prominent. Withers, shoulders and neck structures faintly discernable
3 Thin	Fat build up about halfway on spinous processes, transverse processes cannot be felt. Slight fat cover over ribs. Spinous processes and ribs easily discernable. Tailhead prominent, but individual vertebrae cannot be visually identified. Tuber coxae appear rounded, but easily discernable. Tuber not ischii distinguishable
4 Moderately thin	Negative crease along back. Faint outline of ribs discernable. Tailhead prominence depends on conformation, fat can be felt around it. Tuber coxae not discernable. Withers, shoulders and neck not obviously thin
5 Moderate	Back level. Ribs cannot be visually distinguished but can be easily felt. Fat around tailhead beginning to feel spongy. Withers appear rounded over spinous processes. Shoulders and neck blend smoothly into body
6 Moderately fleshy	May have slight crease down back. Fat over ribs feels spongy. Fat around tailhead feels soft. Fat beginning to be deposited along the side of the withers, behind the shoulders and along the sides of the neck

7 Fleshy	May have crease down back. Individual ribs can be felt, but noticeable filling between ribs with fat. Fat around tailhead is soft. Fat deposited along withers, behind shoulders and along the neck
8 Fat	Crease down back. Difficult to feel ribs. Fat around tailhead very soft. Area along withers filled with fat. Area behind shoulder filled with fat. Noticeable thickening of neck. Fat deposited along inner thighs
9 Extremely fat	Obvious crease down back. Patchy fat appearing over ribs. Bulging fat around tailhead, along withers, behind shoulders and along neck. Fat along inner thighs may rub together. Flank filled with fat

** **<u>Endoscopy</u>**

Mucus quantity:

Score Explanation

0/5 none; clean, singular

1/5 little; multiple small blobs

2/5 moderate; larger blobs

3/5 marked; confluent, stream-forming

4/5 large; pool-forming

5/5 extreme; profuse amounts

A figure taken from Gerber et al, 2004 was provided here

Mukus Score (Gerber et al., 2004)

Mucus viscosity:

Score Explanation

0/3 moist mucous membrane

1/3 serous, flowing secretion

2/3 secretion slowly flowing, moving in the respiratory flow

3/3 highly viscous secretion, immobile, adhesive, endoscope trace remains after retraction

Swelling of tracheal septum

Score	Explanation
0/3	clearly visible tracheal rings and narrow tracheal septum
1/3	low-grade swelling of the tracheal septum
2/3	moderate swelling of the tracheal septum or indistinctly defined tracheal rings
3/3	severe bulbous swelling of the tracheal septum and barely recognisable tracheal rings
