# Supplementary material for: Endoscopically assessed mucus parameters in equine asthma: Relationship to clinical history and cytological findings data
Source: Equine Vet J. 2025 Jul 24;58(3):767–78. doi: 10.1111/evj.70002 (PMC13041601; doi:10.1111/evj.70002)

Equine Veterinary Journal BEVA

**Figure S1:** Flow chart of the analyses and the samples used for them. * Analysis: correlation of mucus quantity score or mucus viscosity score with cell proportions, diagnosis, age, BCS, cough, respiratory rate, swelling of tracheal septum, arterial pO2. ** Analysis: association of mucus quantity score or mucus viscosity score with sex, breed, season of sampling, nasal discharge, respiratory pattern.

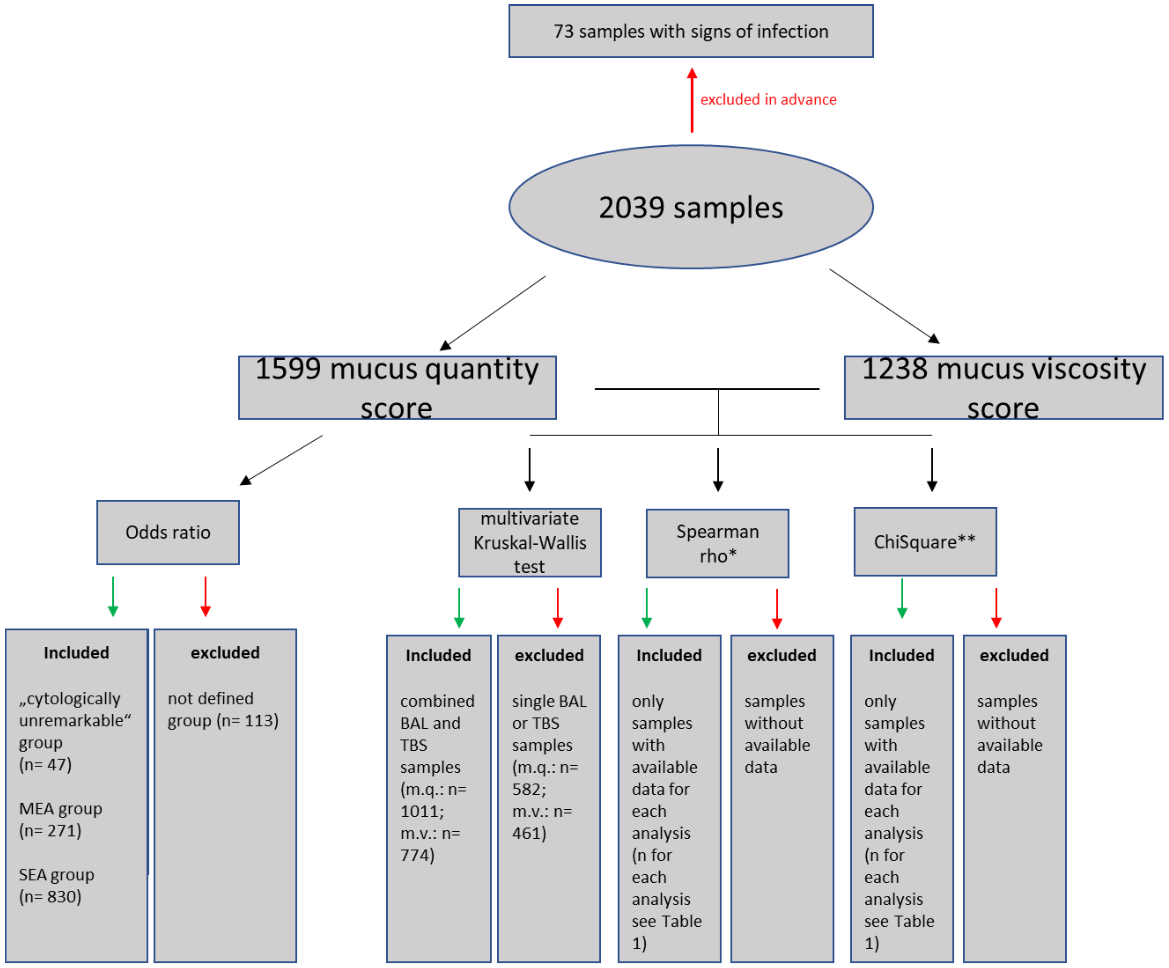

Supplement: Supplementary file 2 — Figure S1. Flow chart of the analyses and the samples used for them. [file EVJ-58-767-s003.pdf]
