# Supplementary material for: Endoscopically assessed mucus parameters in equine asthma: Relationship to clinical history and cytological findings data
Source: Equine Vet J. 2025 Jul 24;58(3):767–78. doi: 10.1111/evj.70002 (PMC13041601; doi:10.1111/evj.70002)
Supplement: Supplementary file 3 — Figure S2. Correlation between BALF cell proportions and mucus quantity and viscosity. [file EVJ-58-767-s009.pdf]

Equine Veterinary Journal BEVA

Figure S2: Correlation between BALF cell proportions and mucus quantity and viscosity scores.

mucus quantity score	mucus viscosity score

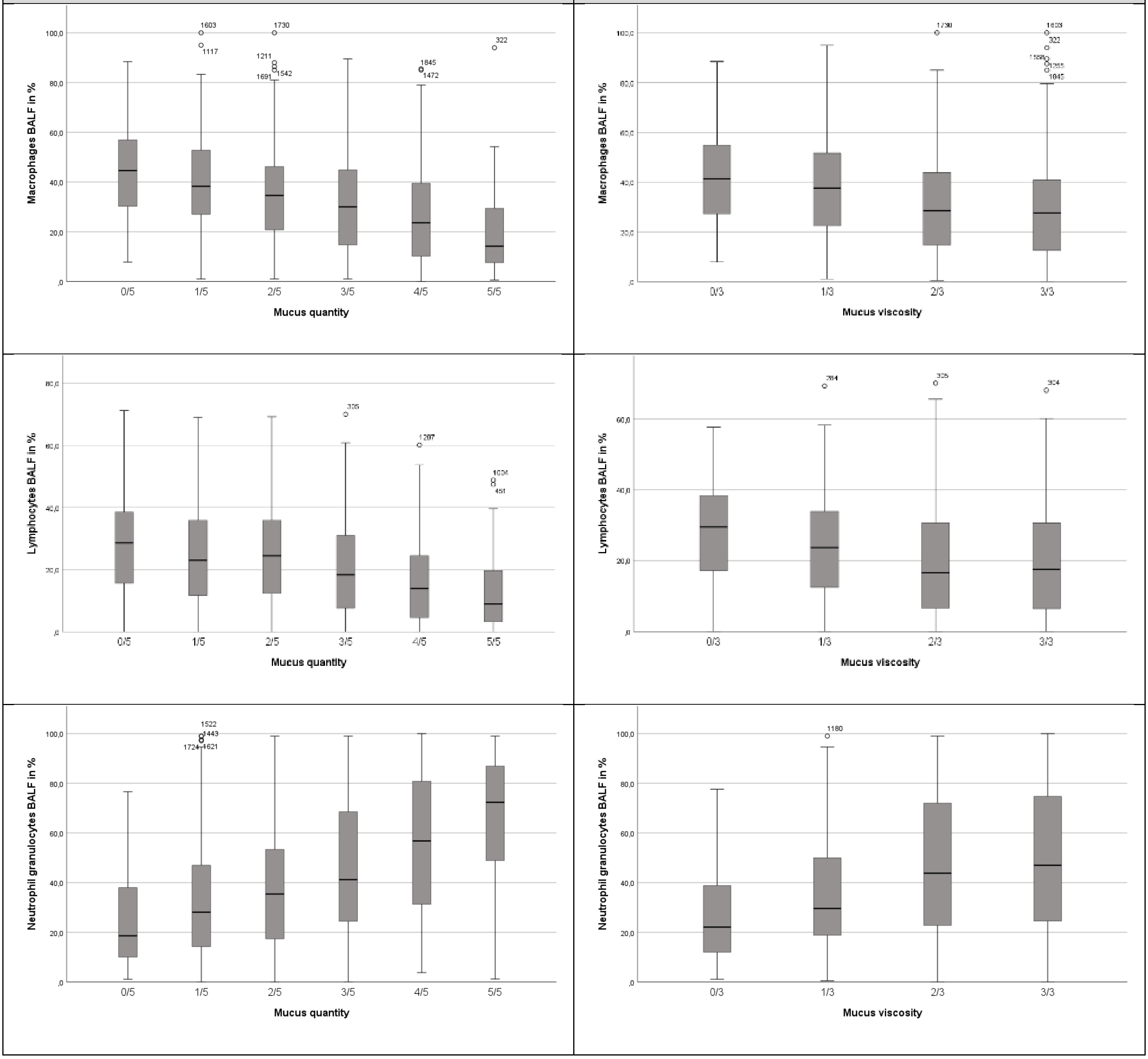

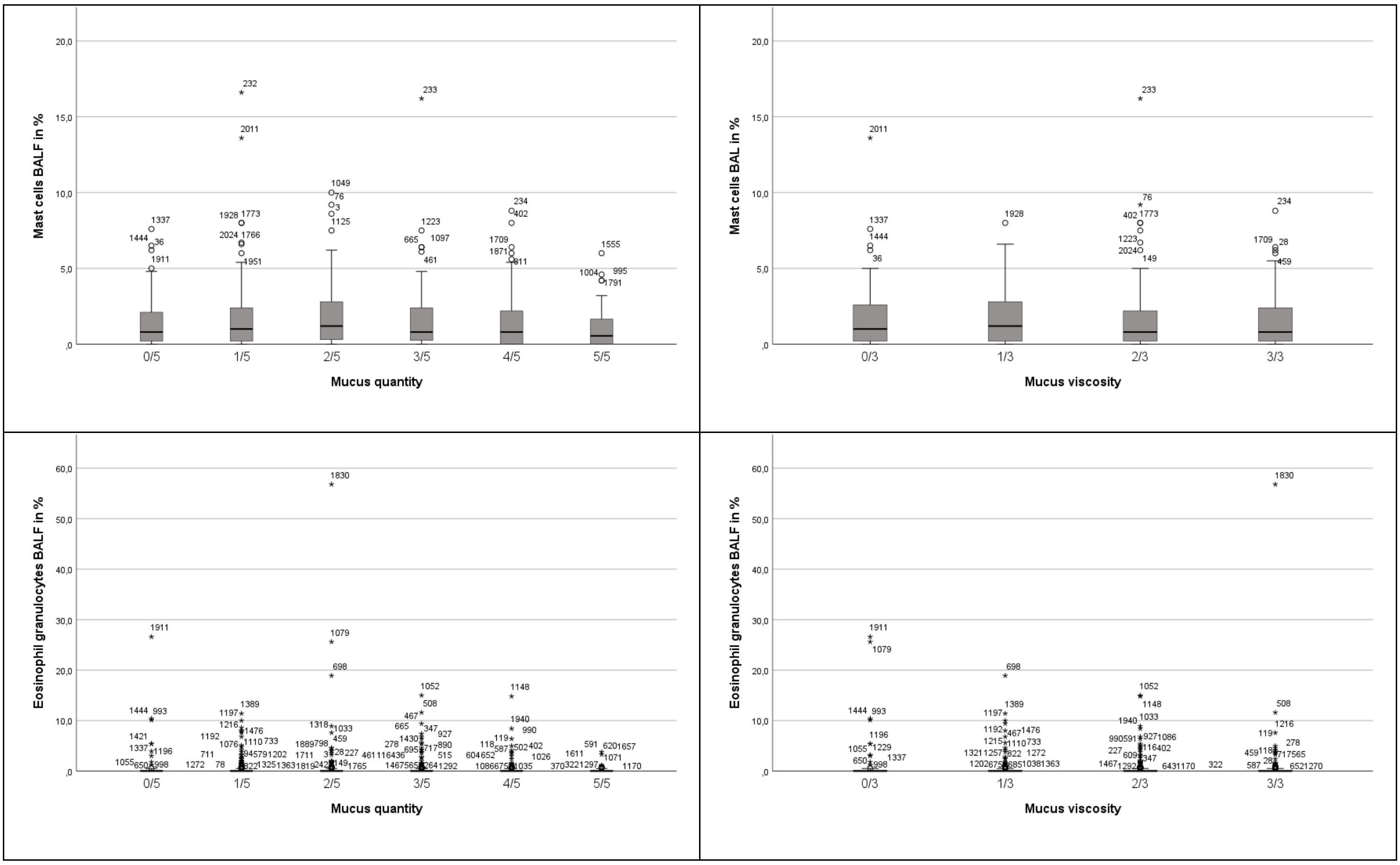

The correlation was significant between mucus quantity score and macrophages (rho= - 0.299; p< 0.001), lymphocytes (rho= - 0.248; p< 0.001) and neutrophil granulocytes (rho= 0.353; p< 0.001). No significant correlation to mast cells (p= 0.756) and eosinophil granulocytes (p= 0.322) was found. Macrophages (rho= - 0.219; p< 0.001), lymphocytes (rho= - 0.143; p< 0.001) and neutrophil granulocytes (rho= 0.225; p< 0.001) were significantly correlated to mucus viscosity score. No significant correlation to mast cells (p= 0.360) and eosinophil granulocytes (p= 0.129) was found. Raw data points are displayed.
