# Supplementary material for: Endoscopically assessed mucus parameters in equine asthma: Relationship to clinical history and cytological findings data
Source: Equine Vet J. 2025 Jul 24;58(3):767–78. doi: 10.1111/evj.70002 (PMC13041601; doi:10.1111/evj.70002)
Supplement: Supplementary file 4 — Figure S3. Correlation between TBS cell proportions and mucus quantity and viscosity. [file EVJ-58-767-s005.pdf]

Equine Veterinary Journal BEVA

Figure S3: Correlation between TBS cell proportions and mucus quantity and viscosity scores.

mucus quantity score	mucus viscosity score
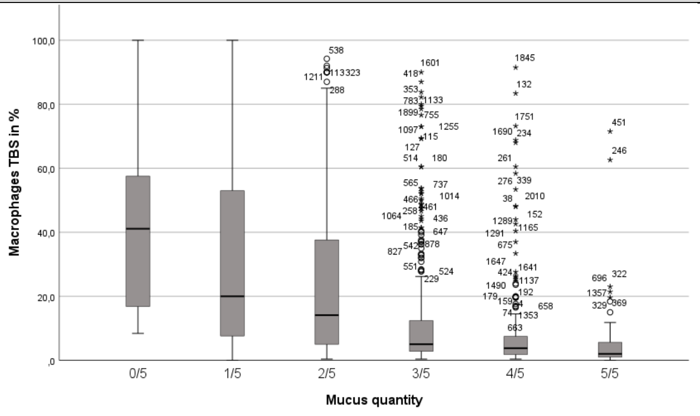	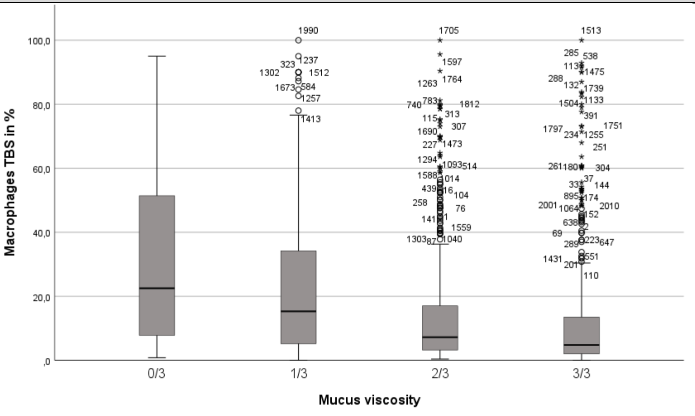
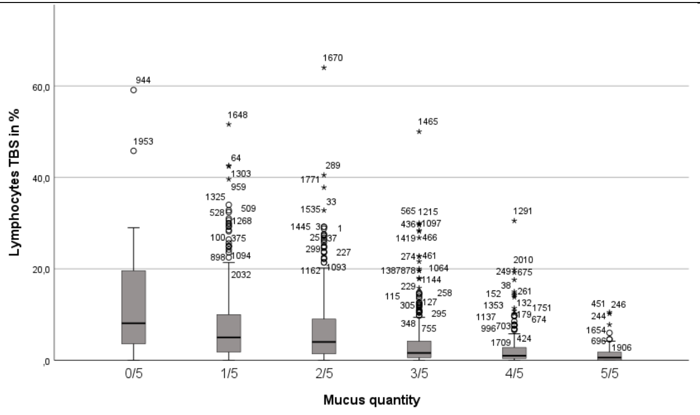	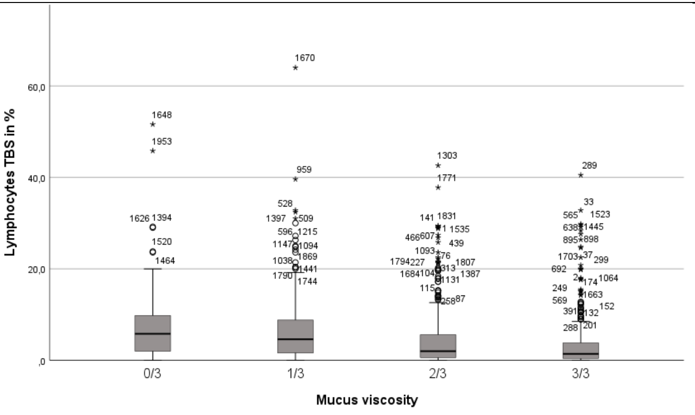
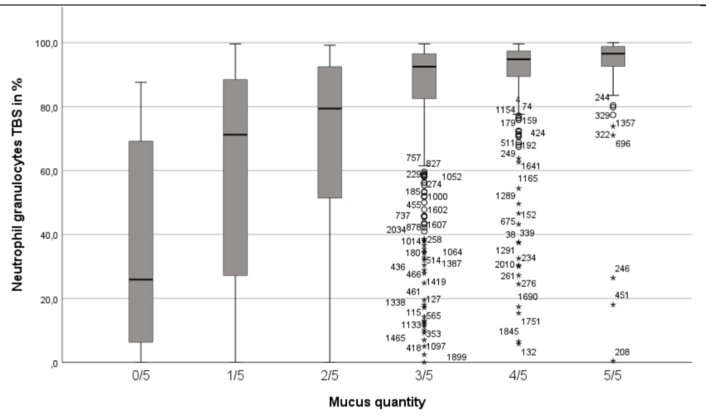	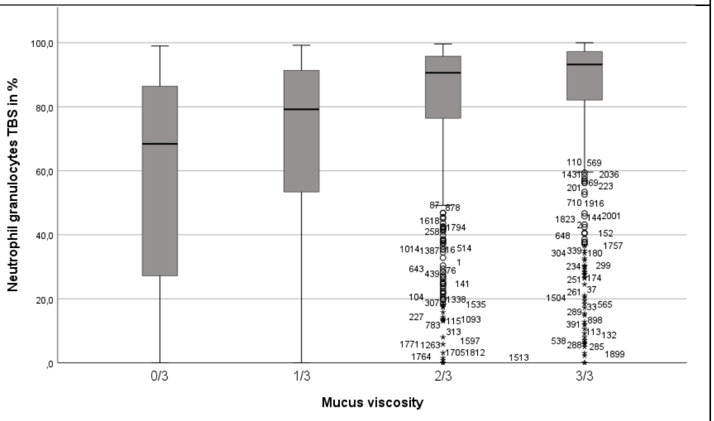

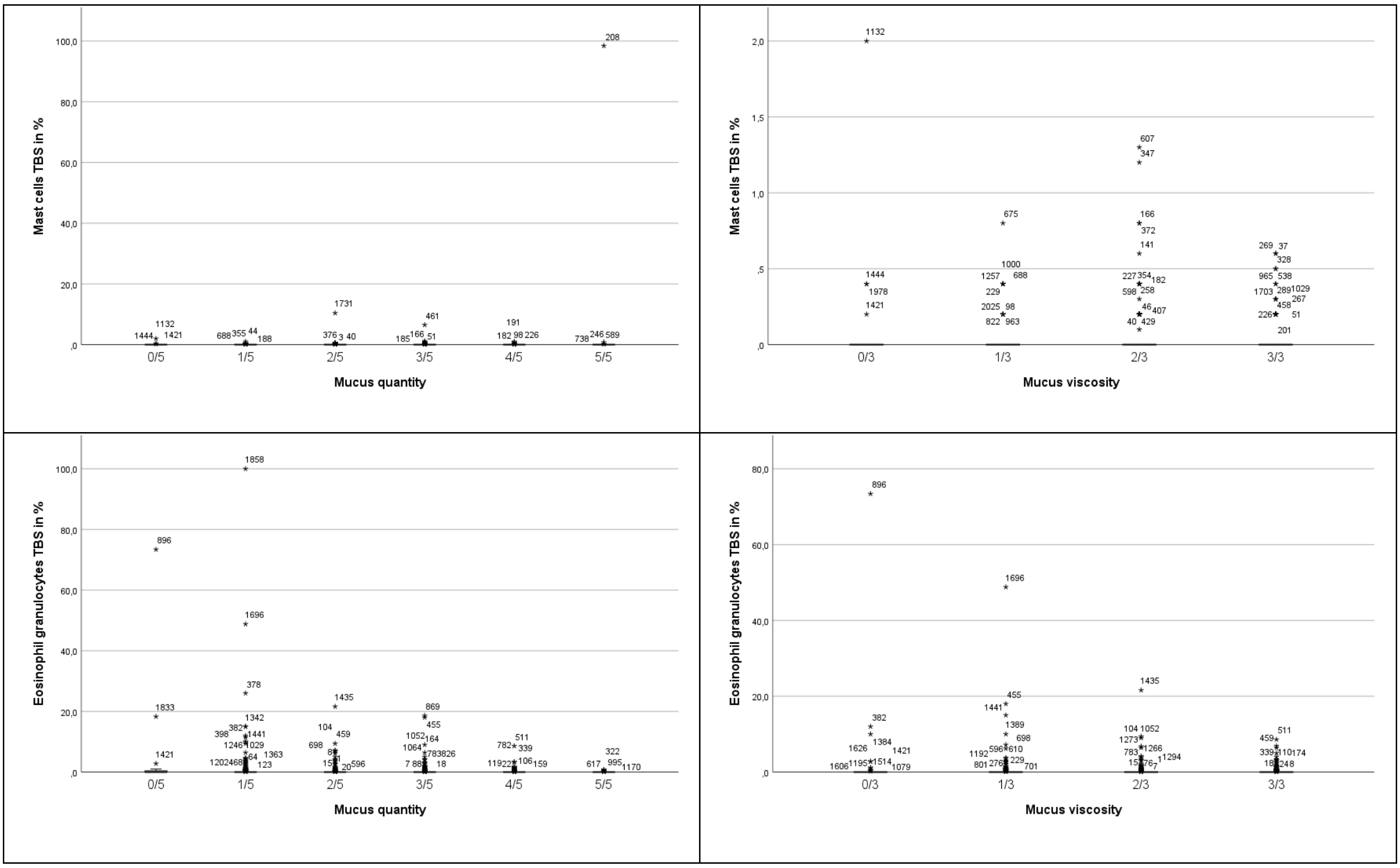

The correlation was significant between mucus quantity score and macrophages (rho= - 0.463; p< 0.001), lymphocytes (rho= - 0.385; p< 0.001), neutrophil granulocytes (rho= 0.477; p< 0.001) and eosinophil granulocytes (rho= - 0.197; p< 0.001). No significant correlation to mast cells (p= 0.366) was found. Macrophages (rho= - 0.254; p< 0.001), lymphocytes (rho= - 0.242; p< 0.001) and neutrophil granulocytes (rho= 0.272; p< 0.001) were significantly correlated to mucus viscosity score. No significant correlation to mast cells (p= 0.360) and eosinophil granulocytes (p= 0.034) was found to mucus viscosity score. Raw data points are displayed.
