# Supplementary material for: Endoscopically assessed mucus parameters in equine asthma: Relationship to clinical history and cytological findings data
Source: Equine Vet J. 2025 Jul 24;58(3):767–78. doi: 10.1111/evj.70002 (PMC13041601; doi:10.1111/evj.70002)
Supplement: Supplementary file 5 — Figure S4. Correlation between mucus quantity/viscosity and age. [file EVJ-58-767-s008.pdf]

Equine Veterinary Journal BEVA

Figure S4: Correlation between mucus quantity and viscosity scores and age.

mucus quantity score	mucus viscosity score
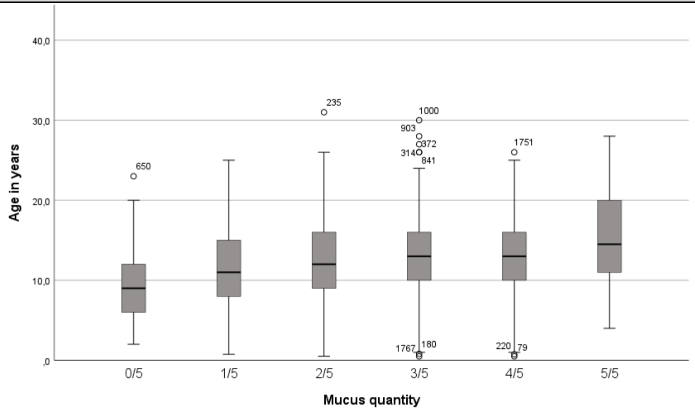	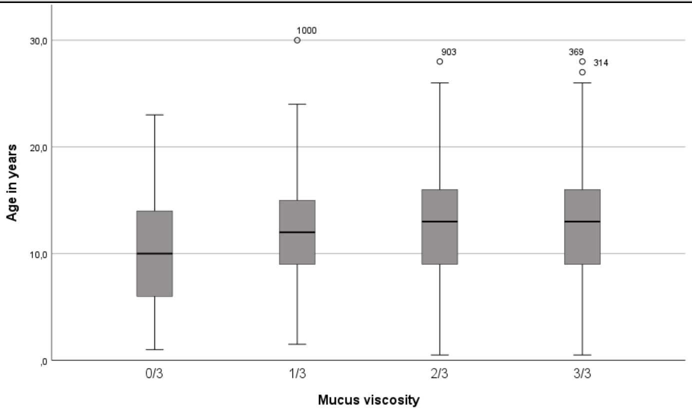

Age was significantly positively correlated with mucus quantity score (rho= 0.190; p< 0.001) and mucus viscosity score (rho= 0.097; p< 0.001). Raw data points are displayed.
