# Supplementary material for: Endoscopically assessed mucus parameters in equine asthma: Relationship to clinical history and cytological findings data
Source: Equine Vet J. 2025 Jul 24;58(3):767–78. doi: 10.1111/evj.70002 (PMC13041601; doi:10.1111/evj.70002)
Supplement: Supplementary file 6 — Figure S5. Correlation between mucus quantity/viscosity and cough, respiratory frequency, swelling of tracheal septum, and pO2 value of arterial blood gas analysis. [file EVJ-58-767-s010.pdf]

Equine Veterinary Journal BEVA

Figure S5: Correlation between mucus quantity and viscosity scores and cough, breathing frequency, septum thickness and pO2 value of arterial bloodgas analysis.

mucus quantity score	mucus viscosity score

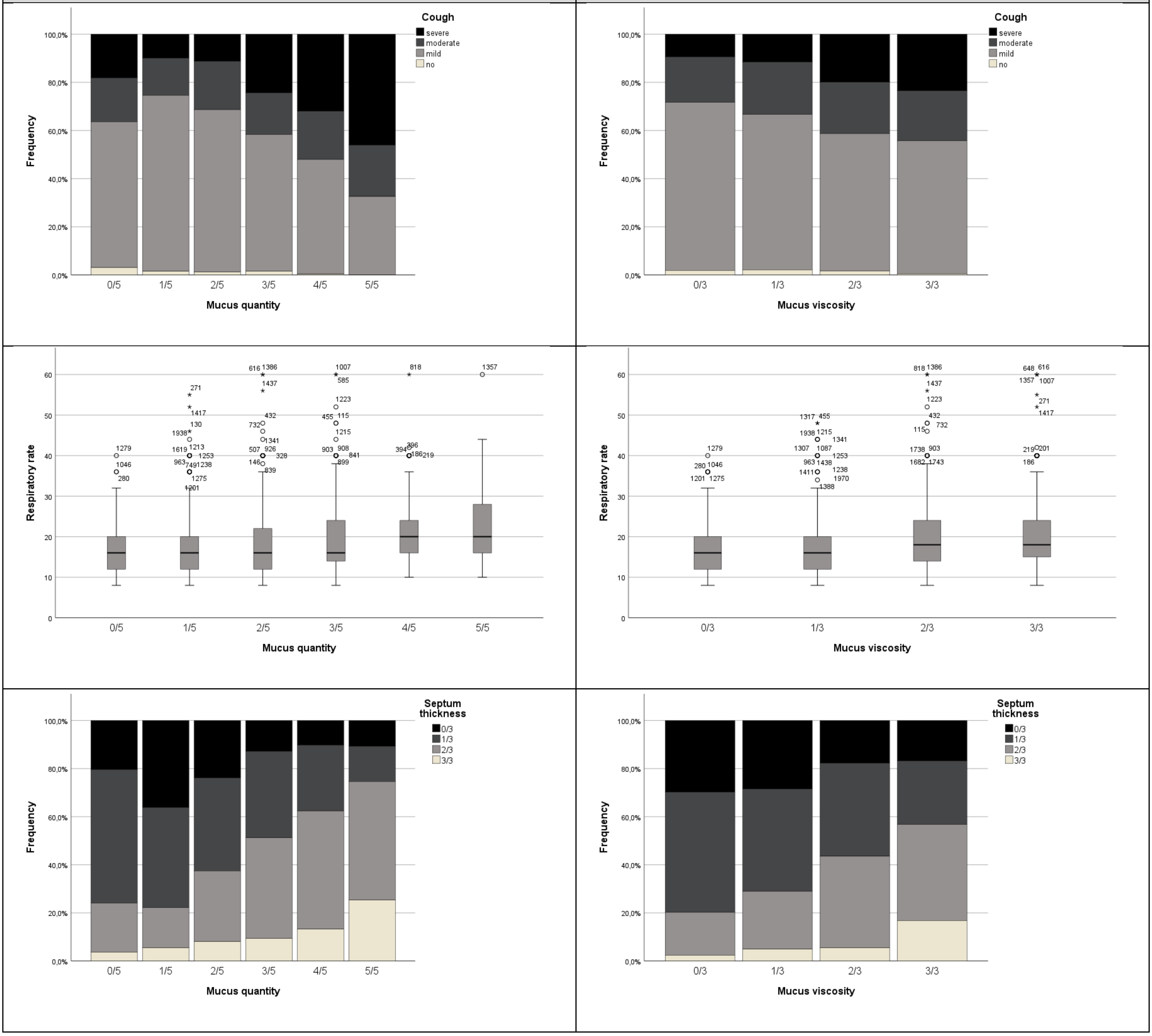

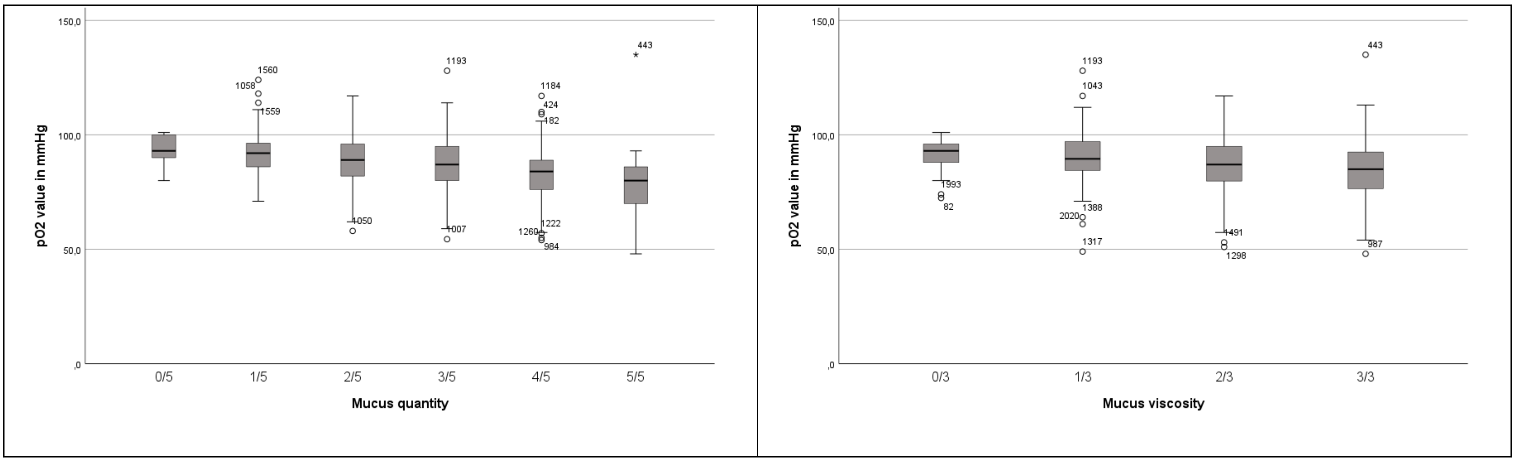

The correlation was significant between mucus quantity score and cough (rho: 0.259; $p < 0.001$), respiratory rate (rho: 0.196; $p < 0.001$), swelling of tracheal septum (rho: 0.328; $p < 0.001$) and arterial pO2 value (rho: - 0.349; $p < 0.001$). Mucus viscosity score was significantly correlated with breathing frequency (rho: 0.138; $p < 0.001$), septum thickness (rho: 0.243; $p < 0.001$) and pO2 value (rho: - 0.207; $p < 0.001$). There was no significant correlation between cough and viscosity (p= 0.042). For clearer presentation, the graphs of the ordinal data (Cough and septum thickness) in the lower graph were scaled to 100%. Raw data points are displayed.
