# Supplementary material for: Endoscopically assessed mucus parameters in equine asthma: Relationship to clinical history and cytological findings data
Source: Equine Vet J. 2025 Jul 24;58(3):767–78. doi: 10.1111/evj.70002 (PMC13041601; doi:10.1111/evj.70002)
Supplement: Supplementary file 7 — Survey S1. Examination questionnaire. [file EVJ-58-767-s002.pdf]

Equine Veterinary Journal BEVA

EquiZyt
Diagnostische Zytologie und Therapieberatung bei respiratorischen Erkrankungen des Pferdes

Date received:_________________

Sample ID: _________________

(completed by the laboratory)

SHIP TO:

EquiZyt UG (haftungsbeschränkt)

Freisinger Str. 8a,

85391 Allershausen, Germany

Tel.: +49 (0) 176 65908331

Email: info@equizyt.com

Part 1: HISTORY (to be filled in by horse owner)

Owner's name: ______________________________ Horse's name: ____________________________

Age: ______________________________ Breed: ____________________________

Sex: O Mare O Gelding O Stallion

Medical History:

Complaint: ____

Cough: O no O mild O moderate O severe

O spontaneous O at beginning of exercise O during exercise O cough attacks

Preceding fever: O no O yes if yes, when: ________________________

Seasonal symptoms: O no O yes if yes, when: ________________________

Discipline and level of performance: ____

Poor performance: O no O mild O moderate O severe

Management:

- **Housing:** O indoor box O open stable
- **Bedding:** O straw O shavings O pellets
  O forest soil O stable mats O others _________________
- **Futter:** O hay O moistened hay O wet hay
  O steamed hay O haylage
- **Riding arena surface:** O sand O sand/textile O textile
- **Other important information:**____

Pre-existing conditions:

O stomach ulcers O wind sucking O PPID (Cushing)

O sweet itch O urticaria (hives) O other skin conditions

Further information:

____

____

____

Part 2: RESULTS OF MEDICAL EXAMS (referring veterinarian) Sample ID: (completed by the laboratory): ______________________________

EquiZyt
Diagnostische Zytologie und Therapieberatung bei respiratorischen Erkrankungen des Pferdes

Referring veterinarian (Stamp or in block capitals):

E-Mail:

Horse's owner: ____________________

Horse's name: ____________________

Date of exam: _____________________

O BAL O TW

Client number: ____________________

Body Condition Score*: O 1 O 2 O 3 O 4 O 5 O 6 O 7 O 8 O 9

Results of clinical exam:

- **Timeline:** O acute O chronic (since: ________________________)
  O chronic with sudden exacerbation
- **Nasal discharge:** O no O serous O mucoid O purulent O sanguineous
- **Breathing pattern:** O unremarkable O increased abdominal effort
  O exspiratory dyspnea O inspiratory dyspnea
  O mixed dyspnea O superficial tachypnea
- **Respiratory rate:**________ /min
- **Cough:** spontaneous: O no O yes cough provocation: O no O yes

Pre-treatment: O yes O no

O Inhaled O Salbutamol O Salmeterol O Budesonide O Beclomethasone O Ciclesonide
O Fluticasone O Ipratropium Bromide

O Orally O Clenbuterol O Acetylcysteine O Bromhexine O Dembrexine O Prednisolone

O Injected O Dexamethasone O others ___________________________________

O Antibiotics O no O yes (_________________________________)

Results of airway endoscopy:

- O pre-training O post-training O with lubricant O with Lidocaine
- **Anomalies in the Pharynx-/ Larynx- region:** ____
- **Tracheal mucus:** O M 0/5 O M 1/5 O M 2/5 O M 3/5 O M 4/5 O M 5/5
- **Mucus viscosity:** O V 0/3 O V 1/3 O V 2/3 O V 3/3
- **Septum thickness:** O S 0/3 O S 1/3 O S 2/3 O S 3/3

Blood gas (pre- / post-exercise):

- **PO2** __________/__________
- **PCO2** __________/__________
- **A-aDO2** __________/__________

Stabilizing agents added to BAL/TW

O no O yes: ___________________

Further information:

____

____

Date and signature (referring vet): ___________________________________
