# Supplementary material for: Endoscopically assessed mucus parameters in equine asthma: Relationship to clinical history and cytological findings data
Source: Equine Vet J. 2025 Jul 24;58(3):767–78. doi: 10.1111/evj.70002 (PMC13041601; doi:10.1111/evj.70002)
Supplement: Supplementary file 8 — Table S1. Scoring of mucus quantity. [file EVJ-58-767-s006.pdf]

Table S1: Scoring of mucus quantity score[11]

Score	Explanation
0/5	none; clean, singular
1/5	little; multiple small blobs
2/5	moderate; larger blobs
3/5	marked; confluent, stream-forming
4/5	large; pool-forming
5/5	extreme; profuse amounts
