# Supplementary material for: Endoscopically assessed mucus parameters in equine asthma: Relationship to clinical history and cytological findings data
Source: Equine Vet J. 2025 Jul 24;58(3):767–78. doi: 10.1111/evj.70002 (PMC13041601; doi:10.1111/evj.70002)
Supplement: Supplementary file 9 — Table S2. Scoring of mucus viscosity. [file EVJ-58-767-s004.pdf]

Table S2: Scoring of mucus viscosity score[29]

Score	Explanation
0/3	moist mucous membrane
1/3	serous, flowing secretion
2/3	secretion slowly flowing, moving in the respiratory flow
3/3	highly viscous secretion, immobile, adhesive, endoscope trace remains after retraction
