# Supplementary material for: Endoscopically assessed mucus parameters in equine asthma: Relationship to clinical history and cytological findings data
Source: Equine Vet J. 2025 Jul 24;58(3):767–78. doi: 10.1111/evj.70002 (PMC13041601; doi:10.1111/evj.70002)
Supplement: Supplementary file 10 — Table S3. Scoring of swelling of tracheal septum. [file EVJ-58-767-s001.pdf]

Table S3: Scoring of swelling of tracheal septum[29]

Score	Explanation
0/3	clearly visible tracheal rings and narrow tracheal septum
1/3	low-grade swelling of the tracheal septum
2/3	moderate swelling of the tracheal septum or indistinctly defined tracheal rings
3/3	severe bulbous swelling of the tracheal septum and barely recognisable tracheal rings
